# The Relationship between Body Mass Index and Post-Cessation Weight Gain in the Year after Quitting Smoking: A Cross-Sectional Study

**DOI:** 10.1371/journal.pone.0151290

**Published:** 2016-03-15

**Authors:** Rebecca A. Krukowski, Zoran Bursac, Melissa A. Little, Robert C. Klesges

**Affiliations:** Center for Population Sciences, Department of Preventive Medicine, University of Tennessee Health Science Center, Memphis, Tennessee United States of America; University of Bremen, GERMANY

## Abstract

**Introduction:**

There is wide variability in the amount of weight gained when quitting smoking, but little is known about key predictors of weight gain. We examined the impact of body mass index (BMI) category and sociodemographic variables on post-cessation weight gain.

**Materials and Methods:**

We utilized National Health and Nutrition Examination Survey data from five consecutive cycles of data collection from 2003–2004 to 2011–2012 to estimate post-cessation weight gain by BMI category among *recent quitters* (n = 654). We analyzed data on their “current weight” and their “past year weight”. We also compared the *recent quitters* with *current smokers*, in order to estimate the amount of weight that could be attributed to quitting smoking.

**Results:**

*Recent quitters* gained 1.4 kg (95% CI: 0.8 to 2.0), while *current smokers* had a non-significant weight change (-0.01 kg (95% CI: -0.3 to 0.2). Weight gain was significant for those in the normal weight (3.1 kg, 95% CI: 2.3 to 3.9) and overweight BMI categories (2.2 kg, 95% CI: 1.1 to 3.2).

**Conclusions:**

BMI category is a key factor in the extent of post-cessation weight gain, with normal and overweight *recent quitters* gaining significant amounts of weight.

## Introduction

While smoking cessation leads to significant improvements in both mortality [1–3] and morbidity [4–6], post-cessation weight gain partially attenuates this benefit on factors such as elevated blood pressure [7] and glucose metabolism [8]. Although the health benefits of smoking cessation clearly outweigh the negative impact of weight gain, concerns about post-cessation weight gain are common and are often cited as a reason to delay cessation attempts [9,10]. In addition, post-cessation weight gain is associated with smoking relapse [9–11].

According to a meta-analysis, post cessation weight gain has been estimated to produce on average, a 1.12 kg, 2.26 kg, 2.85 kg, 4.23 kg, and 4.67 kg weight gain at one, two, three, six, and 12 months after quitting, respectively [12]. However, there is wide variability in the amount of weight gained [12,13]. There is some evidence that those who smoke a greater number of cigarettes [14] and women who are middle aged or older [15] experience larger post-cessation weight gain. Nevertheless, there is limited knowledge in the impact that body mass index (BMI) has on the degree of post-cessation weight gain [16].

We are aware of four studies that examine the impact of BMI category on the amount of post-cessation weight gain, which have conflicting findings [13,17–19]. Travier and colleagues [17] found normal weight men who quit smoking gained more weight than obese men who quit smoking with no other significant associations between BMI category, gender, and post-cessation weight change; however, the follow-up period differed markedly for participants (i.e., between 2–5 years), so weight gain was simply averaged over the time interval, which is unlikely to reflect typical pattern of acute weight gain after cessation, as most of the weight gain would occur in the first six months [12]. In addition, Veldheer et al. [19] reported that normal and overweight former smokers gained the most weight in the 10 years after quitting smoking, while obese former smokers gained the least; however, more proximal weight gain post-cessation was not examined in this study. Lycett and colleagues [13] also examined weight gain among individuals who quit smoking in a clinical trial (N = 85) and reported that obese smokers gained the most weight over eight years compared to other individuals in other BMI categories; however, no estimates on more proximal weight gain was available in this sample. In comparison, Bush et al. [18] found no significant differences in post-cessation weight gain at 6 months by BMI category among individuals who had quit smoking using a quitline. In addition, O’Hara et al. [20] examined the impact of BMI as a continuous variable on post-cessation weight gain; specifically, they found that both men and women with a higher BMI had significantly larger percent weight gain in the first year after quitting. It will be important to clarify characteristics of individuals who may be most likely to gain a substantial amount of weight after quitting smoking, as proximal post-cessation weight gain likely drives relapse due to weight concerns.

Thus, in the present study, we utilized nationally representative data to estimate post-cessation weight gain, both overall and by BMI category. The current study will identify the effect of smoking cessation on weight among *recent quitters* (i.e., smokers who quit within the past year) and compare them with *current smokers*, in order to estimate the amount of weight that could be attributed to quitting smoking. Given the dramatic changes in obesity prevalence over the 25 years of data examined in a meta-analysis by Aubin et al. [12], these analyses also provide insight into contemporary estimates of post-cessation weight gain.

## Materials and Methods

The National Health and Nutrition Examination Survey (NHANES) is conducted by the National Center for Health Statistics of the Centers for Disease Control and Prevention [21]. A nationally representative sample of the non-institutionalized civilian resident population of the United States is selected by means of a complex, multistage probability design. NHANES consists of a computerized in-person interview (which includes questions about demographic characteristics and health) and an examination (which includes physiological measurements and laboratory tests), both of which are conducted by trained examiners. We utilized the data from five consecutive cycles of data collection from 2003–2004 to 2011–2012 (N = 50,912). Over the years, there was an oversampling of various racial and ethnic minority groups and other subpopulations. This oversampling is accounted for via sampling weights, which we use in our analysis along with other design features, for unbiased estimation. The sample was restricted to adults 20 years and older (N = 27,733), as the methodology for collecting smoking data was different for younger individuals. Written informed consent was obtained from all participants, and the protocol was reviewed and approved by the institutional review board at the National Center for Health Statistics.

### Socio-demographic Characteristics

Socio-demographic characteristics were self-reported. Each participant’s race/ethnic group was categorized as non-Hispanic white, non-Hispanic African American, Hispanic, and a category that combined all other race/ethnic groups (due to small sample sizes). Participants were categorized as male or female. Age was reported as a continuous variable and it ranged from 20 to 85 at which point it was truncated for confidentiality reasons. Educational level was classified as less than 12 completed years of schooling, high school graduate/general educational degree or schooling beyond high school. Marital status was categorized as married/living with partner or “single” (which is a combination of never married, divorced, and widowed).

### Weight and Height

Weight at the time of interview and one year ago, as well as current height were self-reported. Participants were categorized according to standard body mass index categories (normal weight: 18.5–24.9 kg/m^2^; overweight: 25.0–29.9 kg/m^2^; obese: 30 kg/m^2^ or higher), using self-reported “past year weight”. Individuals who were categorized as being “underweight” were not included in the analyses, as the sample size was too small for comparisons. Weight for pregnant women was based on pre-pregnancy weight at the time of the interview and one year prior; thus, they were not excluded from the analyses.

Self-reported height and weights were adjusted for reporting error by gender and age group [22]; specifically, we applied a correction for the mean difference between self-reported and measured height by gender, and for self-reported and measured weight by gender and age (i.e., categorical age groups: 18–49, 50–69, and 70 and older), based on NHANES 2001–2006 data. This correction was applied to self-report of both current weight and weight one year ago. Individuals were weighed on a calibrated digital scale as part of the examination component of NHANES; however, as this measured weight is only available for the “current weight” and not the “past year weight,” we chose to use the self-reported weights in order to avoid using weights obtained by different methods, as we would assume that self-report error would similarly impact both of the self-reported weights.

We compared the measured weight with the self-reported weight (adjusted for reporting error [22] correction factors); the mean adjusted self-reported weight for the sample was 81.5 kg (SD = 18.7) and the measured weight was 81.7 kg (SD = 19.7), while unadjusted self-report was 80.9 kg (SD = 18.9). Among men, mean adjusted self-reported weight was 88.4 kg (SD = 18.1) and the measured weight was 88.7 (SD = 19.1), while unadjusted self-report was 88.6 (SD = 18). Finally, among women mean adjusted self-reported weight was 74.8 (SD = 17.5) and the measured weight was 74.9 (SD = 18.5), while unadjusted self-report was 73.5 kg (SD = 17.5).

Among *recent quitters*, we examined their dichotomous response on whether they have tried to lose weight over the past 12 months (yes/no), as this variable may impact the degree of post-cessation weight gain.

### Tobacco Use

We grouped the participants into four categories according to their self-reported tobacco use behaviors: *never smokers*, *current smokers*, *former smokers* and *recent quitters*. Participants who had smoked fewer than 100 cigarettes in their lifetime were categorized as *never smokers*. Participants who reported smoking at least 100 cigarettes in their lifetime and currently smoking cigarettes “every day” or “some days” were categorized as *current smokers*. Participants who reported smoking 100 cigarettes in their lifetime but currently not smoking cigarettes were categorized as either *former smokers* (having quit 1 year ago or greater), or *recent quitters* (having quit between 30 days and 365 days ago). *Recent quitters* who quit 365 days ago were asked to report the number of cigarettes they usually smoked per day before quitting; thus, among this subsample (n = 210), we examined self-reported cigarettes per day that they reported usually smoking per day before quitting, as this factor may influence the degree of weight gain. Some *recent quitters* (n = 230) reported having quit smoking cigarettes yet answered “yes” to a question asking about using nicotine-containing products (e.g., pipes, cigars, chew tobacco, snuff, nicotine patches, nicotine gum); we presume that these participants were dual users prior to quitting smoking cigarettes and continued to use the other product. Given that these participants are still receiving nicotine which could effect weight, we examined the impact of excluding these participants in sensitivity analyses.

### Statistical Analysis

All analyses were performed with SAS, Version 9.4 survey procedures (SAS Institute Inc., Cary, NC, USA). Sampling weights as well as design features such as stratification and clustering were incorporated into the analysis in order to produce unbiased and representative point estimates and their variances. Out of the age restricted sample (n = 27,733 age 20 or older), we excluded 1,977 participants who had missing values for either past year weight, self-reported current weight, with BMI less than 18.5 or greater than 50, current smoking status, or former smokers for whom the time since quitting was missing. Participants excluded due to missing data described above were significantly younger (mean age 44.5 years vs. 46.9 years), more likely to be female (65% vs. 51%), more likely to be of Hispanic descent (27% vs. 12%), more likely to be single (45% vs. 36%), and more likely to have lower education attainment (less than high school degree: 38% vs. 17%).

The final analytic sample was 25,756 participants with complete data. First, we compared the *recent quitters* group with the *current smoker* group, in order to estimate the amount of weight that could be attributed to quitting smoking (versus weight gain over the past year for other reasons, such as overeating over the holidays) as the *recent quitters* were part of the *current smoker* group less than a year ago. Next, we assessed the effect of recent smoking cessation on weight, and we focused solely on the *recent quitters* (n = 654) and the weight change between their current weight and their past year weight. We presented both a crude estimate of weight change as well as an adjusted estimate (i.e., adjusted for baseline weight, time since quitting, age, gender, race, education and marital status). Next, we examined the impact of BMI category, socio-demographic characteristics, time since quitting, the number of cigarettes smoked at the time of quitting, and attempts at losing weight on weight change. Finally, we examined the weight trajectory among *former smokers*, to provide perspective on weight change, after the first year post-cessation.

Comparison of socio-demographic characteristics between categories of smoking behavior were done with a Rao-Scott chi-square test and simple linear regression models for categorical and continuous variables, respectively. Means and their 95% confidence intervals for weight change as well as percent weight change relative to baseline were estimated overall, by baseline BMI category, and for several subgroup combinations that include BMI category, smoking behaviors, weight loss behaviors, and socio-demographic variables. Differences in means between these groups were tested by applying simple linear regression models, which correspond to a t-test and/or analysis of variance, depending on the number of group levels. Multivariable linear regression models were used to test the associations between the weight change and all of the covariates, which included baseline BMI category, smoking behavior groups, socio-demographic variables, all adjusting for baseline weight among *recent quitters*. We present the full model which includes all of the covariates listed above, and the reduced model which retained only significant covariates at p<0.05 and meaningful confounders for adjustment purposes at p<0.1.

We then conducted a few additional analyses, including: (a) a sensitivity analysis of *recent quitters* who reported having quit smoking cigarettes but used a product containing nicotine in the past 5 days, (b) a subgroup analysis of weight change among *recent quitters* with number of cigarettes prior to quitting, and (c) an analysis of weight change among *former smokers* dependent on how long ago they quit. Descriptive, univariate and multivariable methods described above were applied to these additional analyses. All differences and associations were considered significant at the alpha level of 0.05.

## Results

**Fig 1:**
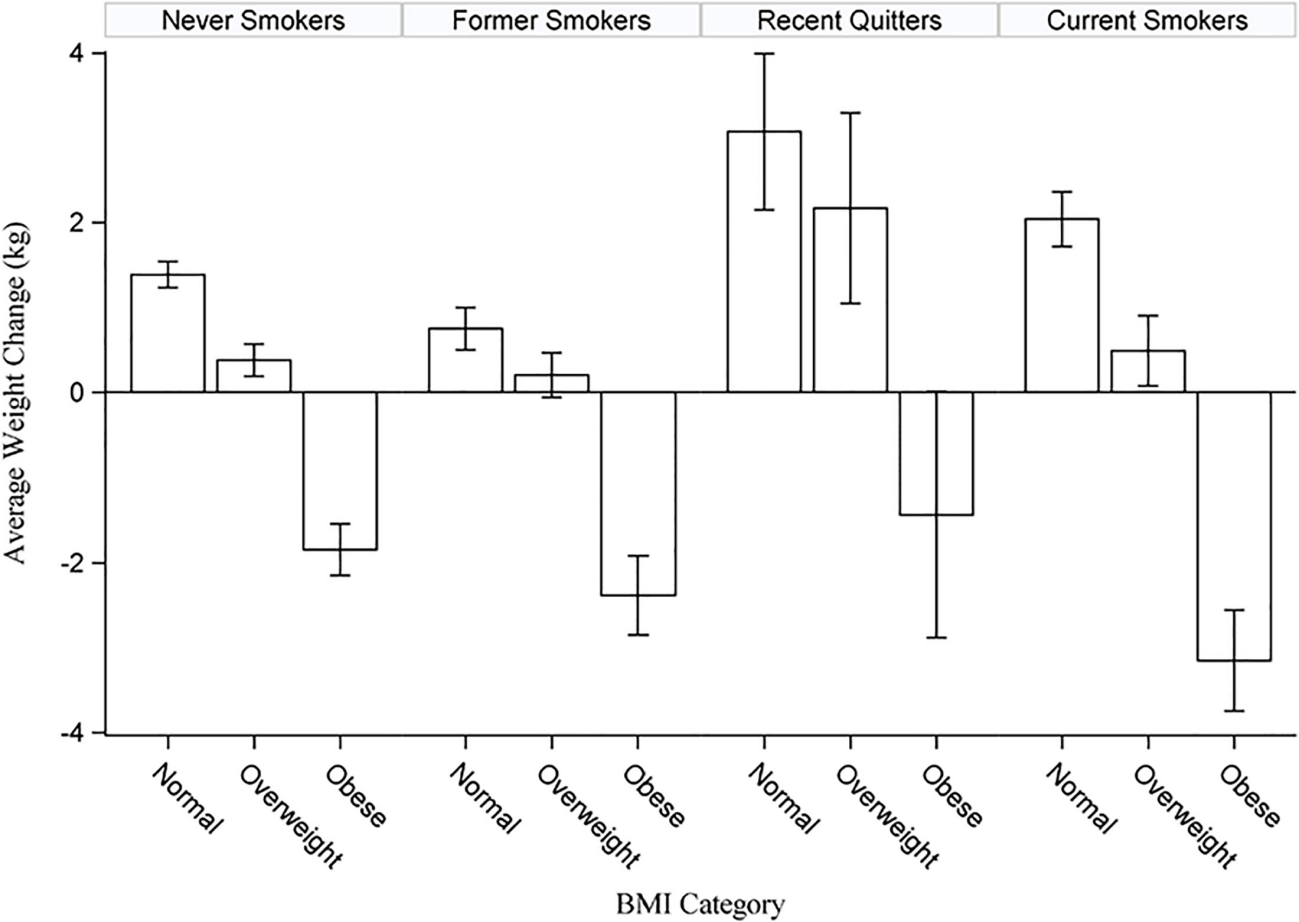
Weight Change by Body Mass Index Category and Smoking Status Group.

Descriptive socio-demographic characteristics of the participants are shown in Table 1 (*recent quitters* n = 654, *never smokers* n = 13,724, *current smokers* n = 5,598, *former smokers* n = 5,780). We also present weight change by BMI category and smoking status group graphically in Fig 1.

**Table 1:** Demographic Characteristics of the Sample by Smoking Status.

	Overall	Never Smokers	Former Smokers	Recent Quitters	Current Smokers
	(N = 25,756)	(n = 13,724)	(n = 5,780)	(n = 654)	(n = 5,598)
*Body Mass Index (%)*					
Normal	33	33	26	37	39
Overweight	34	34	38	33	31
Obese	33	33	36	30	30
*Male (%)*	49	42	56	54	56
*Race/Ethnicity (%)*					
Non-Hispanic African American	11	12	7	9	13
Hispanic	12	14	8	13	11
Non Hispanic White	71	66	81	74	71
Other Race/Ethnicity	6	8	4	4	5
*Married/Partnered (%)*	64	64	71	61	55
*Education Level (%)*					
Greater Than High school Degree	17	14	17	18	26
Less Than High School Degree	24	21	24	23	31
High School Degree or General Educational Development Test credential	59	65	59	59	44
*Age*	47 (0.3)	45 (0.3)	56 (0.4)	40 (0.7)	42 (0.2)

Notes: Proportions presented for all variables with the exception of age, which is presented as mean (SD).

### Weight Change Attributable to Quitting Smoking

In order to estimate the amount of weight that could be attributed to quitting smoking, we compared weight change over the past year among *recent quitters* and *current smokers* based on BMI category as well as gender, race/ethnicity, education level, and marital status. Overall, we found that while *current smokers* had a non-significant weight change (-0.01 kg; 95% CI: -0.3 to 0.2), *recent quitters* gained a significant amount of weight (1.4 kg; 95% CI: 0.8 to 2.0), resulting in a significant difference between these two groups (*p*<0.0001). Over the past year *former smokers* lost a significant amount of weight (-0.59; 95% CI: -0.83 to -0.35) while *never smokers* had a non-significant weight change (-0.03; 95% CI -0.18 to 0.12).

We found significant differences in weight change by BMI category, in addition to significant differences based on socio-demographic characteristics (Table 2). Specifically, we found that, for both *recent quitters* and *current smokers*, normal and overweight individuals reported significant weight gain, and among the current smokers, obese individuals reported a significant weight loss. With the socio-demographic comparisons, notable differences were obese *recent quitters* who were men indicated that they gained a significant amount of weight, while obese *recent quitters* who were women reported no significant change in weight. In addition, *recent quitters* in the “other” race/ethnicity category reported no significant change in weight, regardless of BMI category. Finally, *recent quitters* who were normal weight and had less education than a high school degree reported the largest weight change (5.0 kg).

**Table 2:** Weight Change (in kilograms with 95% confidence intervals) in the Past Year among Recent Quitters and Current Smokers by BMI Category.

	Recent Quitters (n = 654)			Current Smokers (n = 5,598)		
	Normal	Overweight	Obese	Normal	Overweight	Obese
Overall	**3.1 (2.3, 3.9)**	**2.2 (1.1, 3.2)**	-1.4 (-2.9, 0.1)	**2 (1.7, 2.3)**	**0.5 (0.1, 0.9)**	**-3.2 (-3.8, -2.5)**
*Gender*						
Male	**3.0 (1.9, 4.1)**	**1.9 (0.7, 3.2)**	**-2.3 (-4.3,-0.3)**	**1.8 (1.5, 2.2)**	**0.7 (0.1, 1.2)**	**-3.6 (-4.4, -2.9)**
Female	**3.2 (1.9, 4.4)**	**2.6 (0.7, 4.5)**	-0.3 (-2.6, 2.0)	**2.3 (1.7, 2.9)**	0.2 (-0.4, 0.8)	**-2.7 (-3.6, -1.8)**
*Race/Ethnicity*						
Non-Hispanic White	**3.2 (2.2, 4.2)**	**2.1 (0.9, 3.3)**	-1.1 (-2.9, 0.8)	**2.0 (1.7, 2.4)**	0.3 (-0.3, 0.8)	**-3.4 (-4.3, -2.5)**
Non-Hispanic African American	**3.4 (1.3, 5.4)**	**2.9 (0.9, 5.0)**	**-3.6 (-6.3,-1)**	**2.4 (1.6, 3.3)**	**1 (0.3, 1.7)**	**-3 (-4.0, -2.1)**
Hispanic	**3.3 (1.8, 4.8)**	2.2 (-0.1, 4.6)	-2.8 (-5.7, 0.2)	**2.3 (1.5, 3.0)**	**1.5 (0.6, 2.4)**	**-1.7 (-2.9, -0.6)**
Other	0.1 (-0.8, 1.0)	0.8 (-1.8, 3.4)	2.9 (-5.6, 11.5)	**1.3 (0.2, 2.4)**	-0.2 (-1.9,1.4)	**-3.3 (-5.5, -1.1)**
*Education Level*						
Greater Than High School Degree	**2.8 (0.2, 5.4)**	**2.7 (0.8, 4.5)**	-0.5 (-3.6, 2.6)	**2.4 (1.8, 3.1)**	**0.9 (0.2, 1.6)**	**-2.9 (-4.0, -1.9)**
Less Than High School Degree	**5.0 (3.5, 6.5)**	**2.8 (0.8, 4.9)**	0.6 (-2.8, 4.0)	**2.7 (2.1, 3.2)**	0.5 (-0.1, 1.2)	**-2.1 (-3.0, -1.2)**
High School Degree or General Educational Development Test credential	**2.5 (1.4, 3.6)**	**1.8 (0.4, 3.2)**	**-2.8 (-4.3,-1.2)**	**1.4 (1.1, 1.8)**	0.2 (-0.5, 0.9)	**-4.1 (-5.0, -3.2)**
*Marital Status*						
Married/ Partnered	**3.1 (1.9, 4.2)**	**2.3 (1.1, 3.5)**	-1.2 (-2.9, 0.4)	**1.9 (1.5, 2.2)**	**0.7 (0.2, 1.1)**	**-2.3 (-3.0, -1.6)**
Single	**3.1 (1.8, 4.4)**	**1.9 (0.4, 3.5)**	-1.8 (-4.6, 1.1)	**2.2 (1.7, 2.7)**	0.2 (-0.6, 1.0)	**-4.3 (-5.4, -3.2)**

Notes: Bold type indicates statistical significance at *p* < 0.05.

### Effect of Smoking Cessation Over the Past Year on Weight Among *Recent Quitters*

To assess the effect of recent smoking cessation on weight, we focused solely on *recent quitters* (n = 654) and the weight change between their current weight and their past year weight. These individuals had quit smoking, on average, 213 days ago (95% CI: 200 to 226). Overall, they had gained a statistically significant amount of weight, 1.4 kg (95% CI: 0.8 to 2.1 kg), or 2.3% of their baseline weight. After adjusting for baseline weight, time since quitting, age, gender, race, education and marital status, the overall weight gain estimate among *recent quitters* was largely the same (1.4 kg, 95% CI: 0.9 to 2.0).

Broken down by BMI category, individuals in the normal and overweight BMI ranges reported significant mean weight gains (Table 2); individuals in the obese BMI range reported non-significant weight loss. Overall, among *recent quitters*, women reported a significant mean weight gain of 2 kg (95% CI: 1.0 to 3.0). Men reported a significant mean weight gain of 0.9 kg (95% CI: 0.04 to 1.8). Mean weight changes over the past year by BMI category and socio-demographic characteristics are presented in Table 2.

Among *recent quitters* who answered the question about whether they have attempted to lose weight in the past year (n = 575), those who reported that they had tried to lose weight had a similar weight gain (3.5 kg, 95% CI: 2.5 to 4.5 kg) compared to those who reported that they had not tried to lose weight (2.4 kg, 95% CI: 1.7 to 3.2 kg). When broken down by BMI category, normal weight individuals who had tried to lose weight reported a weight gain of 3.5 kg (95% CI: 2.2 to 4.8 kg), overweight individuals who had tried to lose weight reported a weight gain of 4.5 kg (95% CI: 2.9 to 6.1 kg), and obese individuals who had tried to lose weight reported a weight gain of 2.8 kg (95% CI: 0.9 to 4.7 kg). In contrast, normal weight individuals who had not tried to lose weight reported a weight gain of 3.1 kg (95% CI: 2 to 4.1 kg), overweight individuals who had not tried to lose weight reported a weight gain of 3 kg (95% CI: 1.8 to 4.3 kg), and obese individuals who had not tried to lose weight reported a non-significant weight loss of 1 kg (95% CI: -3.6 to 1.6 kg).

For the subsample of *recent quitters* who answered the question about how many cigarettes they smoked prior to quitting (n = 210), we examined this variable in a subgroup model. We found that with each additional cigarette smoked, individuals gained a significant greater amount of weight (i.e., 0.13 kg) over the past year. Based on the BMI category, with each additional cigarette smoked, normal and overweight individuals gained a significant amount of weight (0.13 kg and 0.32 kg, both p<0.0001). In contrast, obese individuals were not significantly effected by the number of cigarettes smoked, with a 0.01kg gain per additional cigarette smoked (p = 0.6).

Some *recent quitters* (n = 230 of 654) reported using a non-cigarette nicotine-containing product (e.g., pipes, cigars, chew tobacco, snuff, nicotine patches, nicotine gum) in the past 5 days. Thus, we conducted a sensitivity analysis to examine the impact of removing these participants from our sample. In comparison to other analyses, we found similar patterns of weight change, overall (1.2 kg, 95% CI: 0.5 to 1.9), and by BMI category (normal weight: 2.9 kg, 95% CI: 2.2 to 3.6; overweight: 2.2 kg, 95% CI: 1.1 to 3.2; obese: -1.6 kg, 95% CI: -3.2 to -0.1).

Finally, we examined the impact of time since quitting on post-cessation weight gain, in order to provide perspective on the weight gain trajectory. We found that among those who quit within last 3 months, weight remained virtually unchanged 0.1 kg (95% CI -1.1 to 1.3), but those who quit 3–6 months ago gained 1.9 kg (95% CI: 0.7 to 3.1), those who quit 6–9 months ago had a non-significant gain of 1.9 kg (95% CI: -0.7 to 4.5), and those who quit 9–12 months ago gained 1.9 kg (95% CI: 0.9 to 3.0).

### Multivariate Analyses Predicting Weight Change By BMI Category Among *Recent Quitters*

Table 3 presents the results of the multivariate linear regression, modeling associations with weight change among *recent quitters* based on BMI category. The full model adjusted for baseline weight, number of days since quitting, age, gender, race/ethnicity, education level, and marital status. In addition, we examined a reduced model that included only those variables that were significant at the *p*<0.1 level as well as BMI category. Using obese individuals as the reference group, normal weight individuals demonstrated a borderline non-significant trend for weight gain (2.6 kg, SE = 1.4, p = 0.06) and overweight individuals had a significant weight gain (2.6 kg, SE = 1.1, p = 0.02). In addition, we found that greater time since quitting smoking and having a high school degree (in comparison to greater than high school education) were significantly associated with greater weight gain. Greater weight one year ago was associated with lower weight gain.

**Table 3:** Multivariate Regression Model Predicting Weight Change among Recent Quitters (n = 654).

	Full Model		Reduced Model^a^	
	Beta (SE)	p-value	Beta (SE)	p-value
*Body Mass Index Category*				
Obese	Ref		Ref	
Normal	2.6 (1.4)	0.0612	2.5 (1.3)	0.0556
Overweight	2.6 (1.1)	0.0205	2.5 (1.1)	0.0214
*Baseline Weight*	-0.02 (0.02)	0.1312	-0.03 (0.01)	0.0621
*Number of Days Since Quit*	0.004 (0.002)	0.0596	0.004 (0.002)	0.0733
*Age*	0.01 (0.02)	0.7309		
*Gender*				
Male	Ref			
Female	0.4 (0.8)	0.6114		
*Race/Ethnicity*				
Non-Hispanic White	Ref			
Non-Hispanic African American	-0.4 (0.7)	0.6248		
Hispanic	-1.1 (0.8)	0.1463		
Other	-0.1 (1.6)	0.9652		
*Education Level*				
Greater Than High School Degree	Ref		Ref	
Less Than High School Degree	1.2 (0.8)	0.1432	1 (0.8)	0.2019
High School Degree or General Educational Development Test credential	2.4 (0.9)	0.0062	2.3 (0.8)	0.007
*Marital Status*				
Single or Other	Ref			
Married/Partnered	0.03 (0.6)	0.9639		

^a^The Reduced Model included only those variables that were significant at 0.01 level in the Full Model.

### Weight Change Among *Former Smokers* Over Ten Years

**Fig 2:**
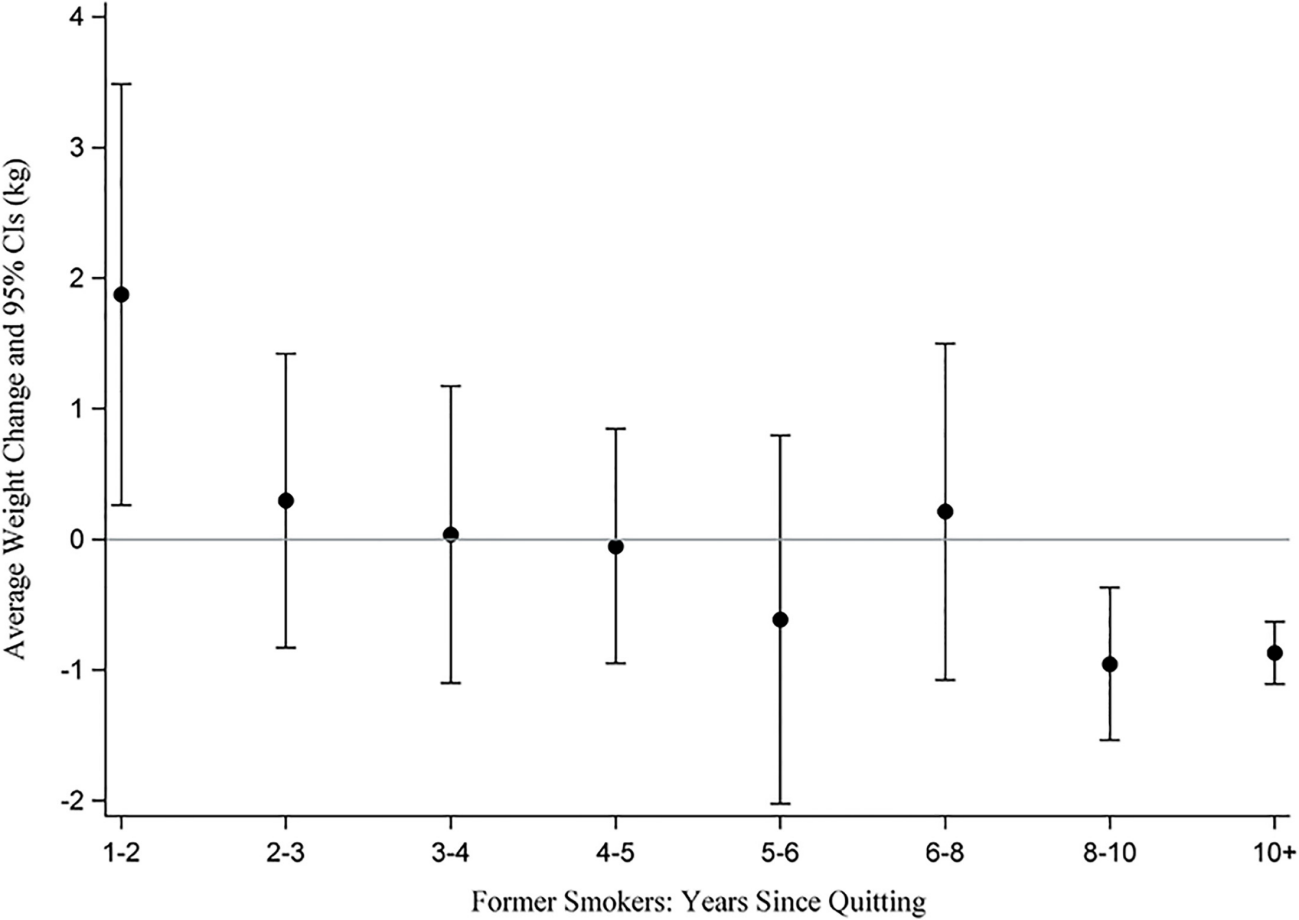
Weight Trajectory Among *Former Smokers*.

In order to give a longer-term perspective on weight change post-cessation, we examined weight change among *former smokers* (i.e., those who quit more than a year ago) in Fig 2. Interestingly, we found significant weight gain between year 1 and year 2, weight stability until year 8, and significant weight loss in years 8 and beyond.

## Discussion

The results of the current study indicate that the weight gain attributable to smoking cessation over a year is approximately 1.4 kilograms; however, consistent with previous studies [12,13], the variability of weight gain by sociodemographic characteristics was considerable. Additionally, post-cessation changes in body weight were influenced by BMI category. In particular, normal weight and overweight individuals gained significant amount of weight after quitting smoking, while obese individuals reported losing weight, though this change was non-significant.

We found that weight gain in the first year following smoking cessation (i.e., 1.4 kg) to be smaller than previous estimates from national population data (2.3 kg among men; 3.1 kg among women) observed by Williamson et al. [23]. These national estimates are also less than an estimate from a meta-analysis of clinical trials (4.7 kilograms) of typical post-cessation weight gain [12]. This is potentially good news for the millions of smokers who attempt to quit smoking each year, as a 1.4 kilogram gain may be palatable to even the most weight conscious smoker. In addition, we found that after the second year post-cessation, participants did not see additional post-cessation weight gain.

Our results also indicate that while normal weight and overweight individuals gained significant amount of weight post-cessation, obese respondents lost (non-significant) amounts of weight. These findings from an epidemiologic sample are consistent with data from Veldheer et al. [19] and partially consistent with treatment-seeking samples described Travier et al. [17] and inconsistent with those described by Lycett et al. [13] and Bush et al. [18] The reason for this is unclear. The most parsimonious explanation is that the obese are at the extreme upper level of the body weight distribution, and we are simply observing regression to the mean in our analyses. However, it is also possible there is some self-reporting bias in weight, but it is not clear why obese individuals (and not normal weight and overweight individuals) would misreport one weight (past year weight) and not the other (current weight). However, it is possible that obese respondents may have the greatest social pressure to report weight loss over the past year. Furthermore, our analysis compared an adjusted self-reported weight with measured weight, and found concordance between these weights. Future studies should replicate and extend our current findings as well as determine the mechanisms for this weight loss upon smoking cessation in the obese.

Both the strengths and limitations of this study should be acknowledged. In terms of strengths, the current study is the most contemporary national dataset to evaluate the relationship between smoking cessation and body weight. The analysis adjusted for self-reported weight error, compared self-reported weight (adjusted) with actual measured weight, and included a broad sample of quitters (those that quit on their own and those that received treatment). However, the weaknesses of our study must also be acknowledged. While the analyses did adjust for self-reported weight bias, some error could have nonetheless been present in using these self-reported weights, particularly recalling the past year weight. However, recollection of past body weights is considered reasonably valid [24–26]. Second, while the sample was representative of U.S. adults, the sample size for some subgroup estimates (e.g., weight loss attempts, number of cigarettes smoked at the time of quitting) was relatively small and should be interpreted with caution. Third, we excluded 8% of the sample due to missing data on key variables, and there were significant sociodemographic differences between those who were missing data and those who were not, which may limit the generalizability of the findings. Fourth, the dataset does not include details on the method of smoking cessation (e.g., behavioral intervention, nicotine replacement therapy, bupropion); thus, we were unable to look at differences in post-cessation weight gain by treatment type. Fourth, *recent quitters* had quit, on average, 213 days prior to the interview; thus, the estimates reflect post-cessation weight gain over about 7 months. However, according to a recent meta-analysis, the majority of the post-cessation weight gain occurs over the first 6 months [12], which is consistent with our findings that mean weight was largely unchanged in the first three months and then by the sixth month, weight gain had peaked. Finally, while use of other nicotine-containing products could potentially inhibit weight gain and impact our findings, we found no significant impact on our conclusions when we removed these participants from our sample and only 1.8% (n = 12) of *recent quitters* reported use of nicotine replacement therapy during the last 5 days.

In summary, the results of the current study suggest that acute weight gain that is attributable to smoking cessation is only about 1.4 kg in this sample, but considerable variability was present, indicating some people lost weight when they quit smoking while some gained considerable weight. An important factor in the magnitude of post-cessation weight gain was BMI category, with normal and overweight *recent quitters* gaining significant amounts of weight. Future studies may wish to focus on normal weight and overweight smokers when implementing post-cessation weight management programs.
